# Development of Cell-Assembled Human Endomysial-Type Biomatrix Substrate for the Detection of Celiac Disease Autoantibodies

**DOI:** 10.3390/ijms26031012

**Published:** 2025-01-24

**Authors:** Ilma R. Korponay-Szabó, Róbert Király, Judit Gyimesi, Markku Mäki

**Affiliations:** 1 Department of Pediatrics, Faculty of Medicine, University of Debrecen, 4032 Debrecen, Hungary; 2 Celiac Disease Center, Heim Pál National Pediatric Institute, 1089 Budapest, Hungary; gyimesi.gallisz@gmail.com; 3 Department of Biochemistry and Molecular Biology, Faculty of Medicine, University of Debrecen, 4032 Debrecen, Hungary; kiralyr@med.unideb.hu; 4 Faculty of Medicine and Health Technology, Tampere University, 33520 Tampere, Finland; markku.maki@tuni.fi

**Keywords:** endomysial antibodies, transglutaminase antibodies, celiac disease, diagnostic accuracy, monkey esophagus

## Abstract

The endomysial antibody (EMA) immunofluorescent test is a highly specific method to detect disease-specific autoantibodies in celiac disease (CD) by their binding to natural transglutaminase-2 autoantigen in tissue sections, and it is used as a compulsory confirmatory test in the non-invasive diagnosis of CD. The classical EMA substrates are the monkey esophagus and the human umbilical cord. It is increasingly difficult to use these tissues due to ethical concerns and animal welfare regulations. In this study, we developed, in cell culture, an endomysium-type extracellular biomatrix assembled by human umbilical cord vein-derived endothelial cells which binds CD antibodies in a similar pattern as monkey esophagus and has similar macromolecular composition. Evaluating retrospectively and prospectively tested patient cohorts, including 130 CD cases and 105 non-celiac controls, IgA-class celiac antibody detection on the biomatrix was equally specific (100%) as EMA testing on tissues, and had higher sensitivity (95.6% versus 91.2%). Both EMA tests were less sensitive, but more specific than transglutaminase-based ELISA measurements. The decellularization of the biomatrix improved sensitivity, enabled the detection of IgG-class celiac antibodies, and allowed for simple reading without previous training. This easily available cell-assembled biomatrix substrate may replace substrate tissues in diagnostic EMA testing in the future.

## 1. Introduction

Celiac disease (CD) is a systemic autoimmune disorder with T lymphocyte activation to ingested gluten peptides and coupled autoantibody production to the transglutaminase 2 (TG2) autoantigen, leading to the damage of the villous structure in the small intestines [1,2]. Since the antigen presentation occurs at mucosal surfaces, CD antibodies are dominantly produced in the IgA class [3], and they also appear in the circulation as diagnostic biomarkers indicating disease activity. IgG-class antibodies are the diagnostically relevant antibodies in subjects with IgA deficiency [4]. Gluten-directed antibodies on their own have little diagnostic value because these antibodies do not have the needed sensitivity or specificity for CD [3,5].

Celiac autoantibodies can be detected by various immunoassays, of which the most specific is the so-called endomysial antibody (EMA) assay [3,6,7]. This immunofluorescent test offers the celiac autoantigen in its natural context with the exposure of the disease-relevant conformational TG2 epitopes, mainly epitope-2, targeted by approximately 90% of the circulating TG2-directed CD autoantibodies [5]. Traditionally, frozen-cut unfixed sections from a monkey esophagus or human umbilical cord are used, which, after incubation with appropriately diluted serum or plasma samples, bind the celiac antibodies in a distinct pattern along endomysium, the outer sheet of muscular structures. The resulting honeycomb binding pattern is very specific for CD and can be visualized by microscopy by adding anti-IgA secondary antibodies labeled with fluorophores. Earlier studies have shown that the celiac antigen in the endomysium is extracellularly localized TG2 bound to fibronectin [8] and collagens [9]. Sections from primates are needed for appropriate sensitivity because rodent and other animal TG2s have only epitope-1 and other minor epitopes [10], but not the dominant epitope-2 [11].

Although the initial detection and quantitation of anti-TG2 antibodies can be performed on a larger scale by higher throughput or automated methods, such as ELISA or ELIA assays, the obtained numerical results differ between tests from different manufacturers due to the lack of standardization. This explains why only much higher values than the manufacturers’ cut-off reliably predicted villous atrophy in prospective studies [12]. In line with this, the European Society for Paediatric Gastroenterology, Hepatology and Nutrition (ESPGHAN) still requires in its most recent (2020) guidelines IgA TG2 antibody serum concentration over 10 times the upper limit of normal (≥10 × ULN) plus a positive EMA test result from a separately drawn blood specimen for the final CD diagnosis [2] if it is intended to be made with the omission of endoscopy and small bowel histology. This approach tries to minimize two common risks for false life-long diagnosis of CD: the potential mix-up of laboratory samples and non-specific sticking of antibodies to recombinant TG2s, their non-coding tags, or simply to plastic surfaces [13]. It should be emphasized that the celiac epitopes of TG2 are highly dependent on the correct folding, stability, and good quality of the protein needed for specific results [11]. Moreover, the Ca^2+^ -activated extracellular conformation is the preferred antigen for CD antibodies, whereas intracellular TG2 bound to GDP is not well recognized [13,14].

A shortage of EMA tissue section sources has recently emerged since monkeys are an endangered species and the use of possible alternatives, such as human umbilical cord or normal appendix sections [15], is also increasingly restricted by ethical concerns. Earlier attempts to apply fixated cells as antigenic targets [16,17] did not gain application in clinical practice because these systems did not provide extracellular TG2, and cellular components often bound other (non-celiac) antibodies as well.

Our study aimed to produce an extracellular matrix in cell culture with endomysial characteristics and CD antigenicity, thus avoiding the use of human or animal tissues as laboratory testing substrates. We show here that such a biomatrix assembled by human umbilical cord vein-derived endothelial cells (HUVECs) can be utilized even in a cell-free format with high sensitivity and specificity to detect celiac autoantibodies, and can replace the traditionally used tissue sections.

## 2. Results

### 2.1. Development of CD-Antigenic Biomatrix in Cell Culture

#### 2.1.1. Selection of Cell Lines and Antigen Visualization

Umbilical cord-derived fibroblasts, myofibroblasts, and endothelial cells (HUVECs) are known to contain high amounts of TG2 [16,17,18], which, after conventional culturing and fixation with paraformaldehyde, can be recognized by staining with monoclonal TG2 antibodies CUB7402 targeting a linear epitope, but not by CD autoantibodies (Figure S1). It was, thus, concluded that the conformational epitopes of TG2, which are important for celiac antibody binding, did not survive the fixation procedure. When other fixatives, such as acetone or methanol (MES-buffered), and acetone-resistant plasticware were applied, the fibroblasts and myofibroblasts showed prominent CD antibody binding, but mainly in their cytoplasm, with only a minimal externalization of the antigenic TG2 to the extracellular matrix. Although the high fetal bovine serum (FBS) content of the medium promoted the export of TG2 to the matrix, the protein appeared in bunches on the cell surface without a definite pattern (Figure S1). HUVECs, however, started to produce under the cells a very fine spider net-like extracellular structure with CD antigenicity. Therefore, HUVECs were selected for further study and were immortalized by the viral delivery of the telomerase gene using pBABE-neo-hTERT [19] to enable long-term culturing and more reproducibility.

#### 2.1.2. Optimization of the Biomatrix Production

First, we noticed that a more regular CD antigenic extracellular network was produced if the cells were seeded to the plastic chamber slides without coatings by gelatin or fibronectin solutions. These compounds promote cell adhesion and are widely used when culturing HUVECs, but are known to contain or capture TG2. When present on the plate in an amorphous distribution, they interfered with the building-up of the TG2-containing network by the cells, and thus the binding of CD antibodies did not result in a clear visual pattern.

**Figure 1:**
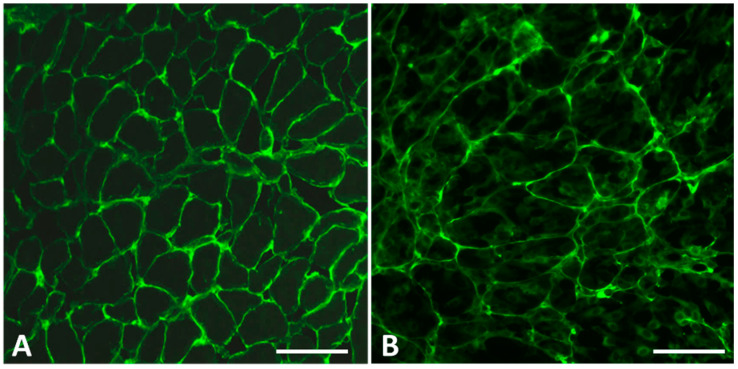
Endomysial antibody binding to tissues and to the cell-assembled biomatrix. (**A**) Monkey esophagus sections incubated with patient serum containing IgA-class celiac disease autoantibodies recognized by fluorescein isothiocyanate-conjugated anti-human secondary antibodies; (**B**) binding of the celiac IgA antibodies to extracellular matrix assembled by human umbilical cord vein-derived endothelial cells. Bars represent 50 µm.

Our goal to let the cells produce a TG2-rich extracellular matrix organized into a well-recognizable and reproducible pattern was achieved by the sequential use of culture media in the following way: (a) immortalized HUVECs were maintained in culture in Medium 199 with 10% FBS and 10% EGM-2 endothelial medium (completed with Single Quots^TM^ additives), (b) seeded to chamber slides in undiluted (100%) EGM-2, and (c) at 24–48 h after seeding, the medium was changed back to Medium 199 with 10% FBS and 10% EGM-2. Under these conditions, the HUVECs produced in 4–6 days a well-organized extracellular matrix (hereafter referred to as HUVEC-ECM), which bound CD IgA antibodies in a similar pattern as the endomysium in tissues (Figure 1).

The experiments presented in Table S1 and Figure S2 showed that this type of biomatrix was less efficiently produced if the same culture medium was used throughout the whole process. Medium 199, complemented with 10% FBS but not with EGM-2, did not promote sufficient cell adhesion and biomatrix production. The use of undiluted EGM-2 for a longer time after seeding resulted in the high intracellular production of TG2 with delayed externalization in a slightly different pattern (Figure S2); hence, the recognition of the CD antibody binding pattern was more difficult and could be disturbed by non-celiac serum antibodies that bind to cell bodies.

**Figure 2:**
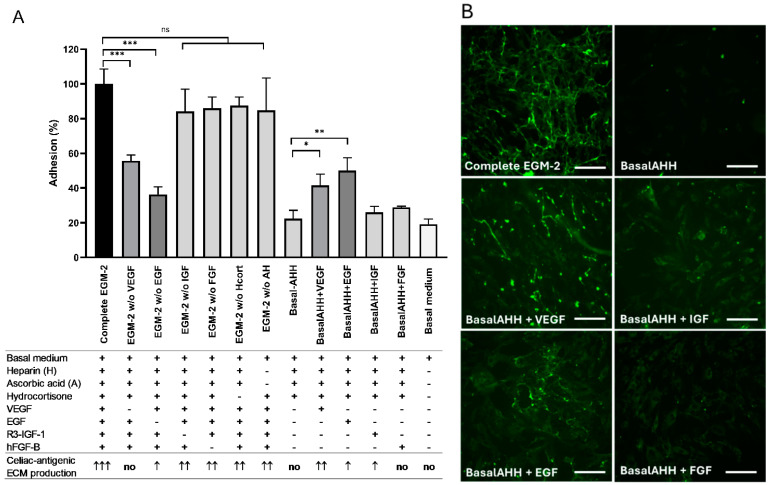
Adhesion and the subsequent production of extracellular matrix network with celiac antigenicity by human umbilical cord vein endothelial cells cultured with and without growth factors. Experiments were conducted with 3 replicate wells for each combination. (**A**) Cell adhesion was quantified at 24 h after seeding by the Image J cell counting analysis tool and expressed as relative to the values obtained in complete EGM-2 medium, consisting of basal medium and all the listed additives. Matrix production was evaluated on the 6th day microscopically as fully (↑↑↑) or partially (↑↑) organized antigenic network recognized by celiac antibodies, patchy positivity (↑), or no positivity; representative images are shown in panel (**B**). Pictures of the experiments when growth factors were added one by one are presented. Bars represent 50 µm. The basal medium was Endothelial cell growth basal medium-2 (Lonza CC-3156) + 2% fetal bovine serum and Penicillin–Gentamicin. ECM, extracellular matrix; VEGF, vascular endothelial growth factor; EGF, human epithelial growth factor; R3-IGF-I, human insulin-like growth factor-I and hFGF-B, human fibroblast growth factor; H, heparin; A, ascorbic acid; Hcort, hydrocortisone. BasalAHH, basal medium with added ascorbic acid, heparin, and hydrocortisone. ns: not significant, * *p* < 0.05, ** *p* < 0.01, and *** *p* < 0.001. Error bars indicate the standard error of the mean.

In the next experiments, we sought to establish which components of the complex EGM-2 medium, which contains seven growth factor additives (Single Quots), are important for cell adhesion and the HUVEC-ECM biomatrix production. We cultured the cells omitting one additive at a time or adding only one additive to the basal EGM-2 fraction of the medium during the (a)–(c) steps of the culturing. The adhesion of HUVECs was poor after 24 h if only the basal medium was used without growth factors and the cells did not produce celiac antigenic ECM by the 6th day. The omission of either heparin, ascorbic acid, hydrocortisone, R3-IGF-I, or hFGF-B did not significantly influence either the adhesion or matrix production. HUVEC-ECM production was enhanced by VEGF even if it was used as a single growth factor in the system, but VEGF only marginally promoted adhesion. EGF promoted adhesion and to some extent, also HUVEC-ECM formation (Figure 2), partly through the higher number of adhered cells and earlier confluency. Thus, both VEGF and EGF seem to play a role in the production of the HUVEC-ECM for celiac diagnostic purposes. However, the optimized composition of the complete EGM-2 medium was more advantageous for cell fitness and consequently, for enhanced biomatrix production.

### 2.2. Molecular Characterization of the Biomatrix

**Figure 3:**
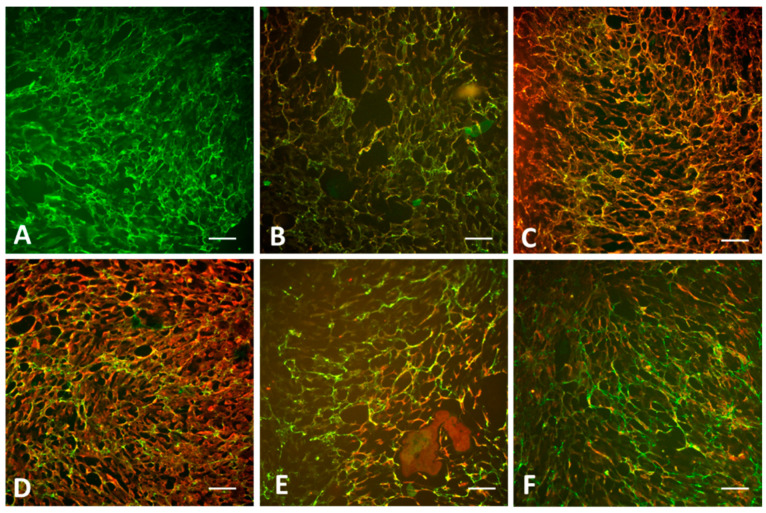
Celiac antibody binding in relation to transglutaminase 2 and other extracellular or cell adhesion proteins in HUVEC-ECM after methanol fixation. Chamber slide wells with cell-assembled biomatrix were stained (**A**) with celiac IgA antibodies, and double-stained with celiac IgA and (**B**) monoclonal antibodies CUB7402 to transglutaminase 2, (**C**) rabbit polyclonal antibodies to fibronectin, (**D**) laminin, (**E**) monoclonal antibodies to vinculin, or to (**F**) VE-cadherin. Human IgA binding was visualized by fluorescein isothiocyanate-conjugated secondary antibodies in green, and rabbit or mouse antibodies were visualized by Alexa Fluor 568-conjugated anti-rabbit or anti-mouse antibodies, as appropriate, in red. Bars represent 50 µm.

The HUVEC-ECM slides were studied by multicolor fluorescent labeling for extracellular, intracellular, or basement membrane proteins and their relation to the observed endomysial-type binding pattern of CD antibodies. For this experiment and further studies on clinical samples, biomatrix specimens were fixated in buffered methanol to better preserve the structure and intracellular proteins that can be achieved by acetone. CD IgA binding (Figure 3A) showed complete co-localization with TG2, partially co-localized with fibronectin and laminin, but did not overlap with cellular antigens, such as vinculin and VE-cadherin (Figure 3B–F), consistent with the knowledge that the antigenic component for celiac antibodies is extracellularly localized TG2 bound to the surface of fibronectin. Cloned antibodies selectively targeting the celiac epitope-2 of TG2 displayed the same binding pattern as shown in Figure 3A. To further prove that the celiac antigen exposed in the HUVEC-ECM is extracellular, we removed the cells from the slides by 0.1% deoxycholate before fixation, which resulted in the lack of DAPI signal. This procedure did not affect either the intensity of the CD antibody binding or the structure of the produced biomatrix.

**Figure 4:**
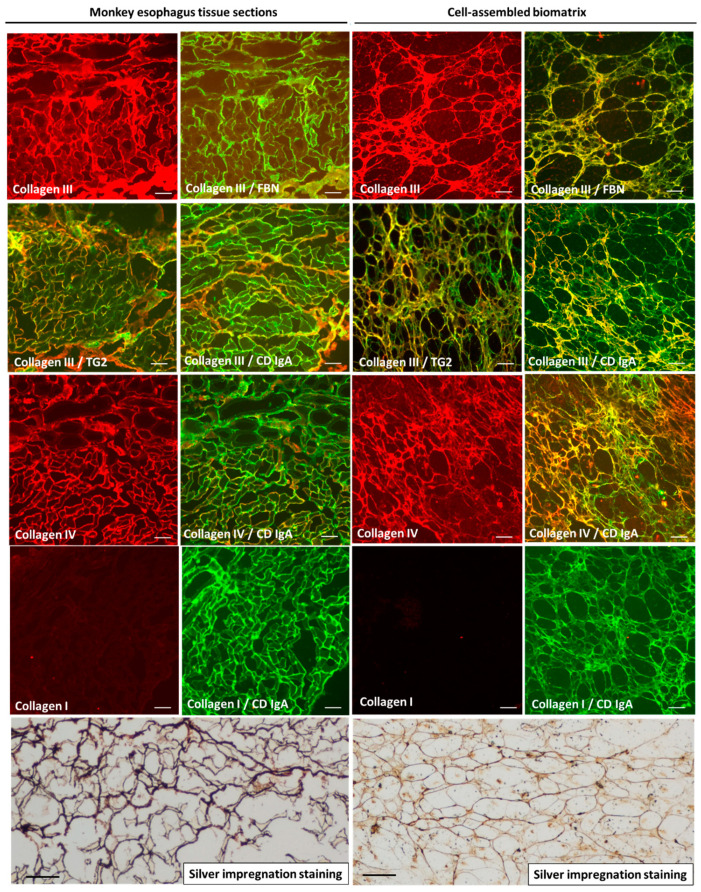
Macromolecular composition and structure of the decellularized biomatrix in comparison with the endomysial structures of a monkey esophagus. Collagens I, III, and IV were detected by monoclonal mouse antibodies and Alexa Fluor 568 secondary antibodies in red; fibronectin (FBN), transglutaminase 2 (TG2), and coeliac IgA antibody (CD IgA) binding were detected by Alexa Fluor 488 secondary antibodies or by FITC-conjugated anti-human IgA in green. The merging of the red and green labels into yellow indicates co-localization. Black staining with the classical silver impregnation method demonstrated that both monkey endomysium and the biomatrix are built up of a similar network of reticulin. Bars represent 50 µm.

The decellularized HUVEC-ECM was used to analyze the macromolecular composition of the biomatrix in comparison with monkey esophagus tissue sections (Figure 4). Monkey esophagus sections and the HUVEC-ECM yielded identical results for fibronectin, collagen III, and IV positivity and distribution patterns, known major components of the endomysium, and both had, similarly, a low expression of collagen I. Moreover, the cell-free biomatrix network was positive with the classical silver staining in the same pattern as is defining endomysial structures in histopathology (Figure 4). Taken together, these results indicate that the produced HUVEC-ECM has an endomysial character which makes it suitable for EMA testing in diagnostics.

### 2.3. Diagnostic Performance in Comparison with Established Clinical Assays in Prospectively Tested Patient Cohort

Serum samples from 90 patients (median age 6.4 years, range 2.4–40) consecutively referred because of celiac antibody positivity (Table S2) were prospectively tested. In the case of the prospective enrollment, the first developed version of the biomatrix containing cells was utilized throughout with 1:10 serum dilutions that correspond to 1:2.5 dilutions on tissue sections.

The transglutaminase antibody clinical ELISA (TGA) results for IgA antibodies were positive (>3 U/mL) at our testing in 73 of the 90 prospectively studied samples, with EMA-IgA on tissues in 62 and EMA-IgA on the HUVEC-ECM in 67 samples. In 17 samples, all three antibodies were negative. In these seronegative patients, the earlier result at the referral site was TGA-IgA ELISA laboratory positivity in four cases, isolated TGA-IgG positivity in seven, deamidated gliadin-peptide (DGP)-based home test positivity in four, and doubtful other test positivity in two. At the time of the present study, none of these patients followed a gluten-free diet, nor did any patients with earlier IgG-class antibody positivity display TGA-IgG or EMA-IgG positivity in our clinical testing, and they were not IgA deficient.

Patients were further evaluated by the ESPGHAN 2020 guidelines (detailed flowchart in Figure S3). Accordingly, the diagnosis of CD was established in 50 patients by the non-invasive route. Twenty-four patients underwent duodenal biopsy, of whom 18 had Marsh II-III histology results, confirming CD in altogether 68 (76%) of the 90 enrolled patients and excluding CD in 6 (6.7%, all these EMA-negative on both the tissues and HUVEC-ECM). The remaining prospectively investigated patients with negative TGA and negative EMA did not have justification for endoscopy.

Table 1 shows the sensitivity, specificity, positive/negative predictive values, and diagnostic accuracies of the three antibody tests in relation to the final diagnosis.

When the EMA results were analyzed in relation to the TGA-IgA concentrations (Table S3), 16 of the 22 samples found low positive in TG2-ELISA showed EMA positivity on the HUVEC-ECM, whereas only 11 on conventional tissues. Thus, EMA testing on the HUVEC-ECM was more sensitive than classical EMA testing.

### 2.4. Decellularization of the Biomatrix Further Improves Sensitivity

In order to evaluate the efficacy of EMA detection on a larger patient material, we tested 62 additional untreated patients with biopsy-proven CD and 105 non-celiac control patients with normal histology utilizing the decellularized version of the biomatrix.

**Figure 5:**
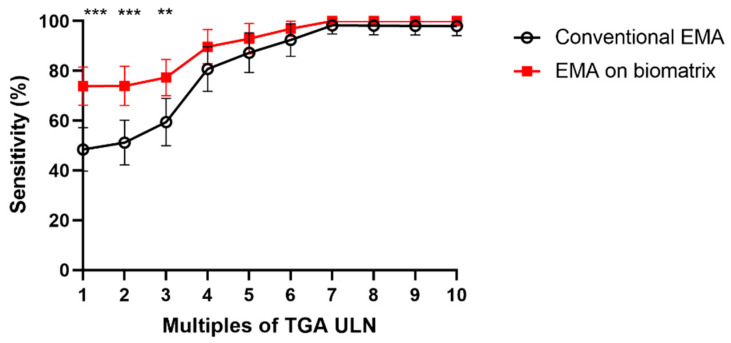
Diagnostic sensitivity of the endomysial antibody (EMA) IgA detection for celiac disease using the biomatrix substrate compared to conventional EMA assay using tissue sections. The results of the celiac disease patients with confirmed diagnosis by histology or according to ESPGHAN non-biopsy criteria (n = 130) are plotted at 1–10 upper limit of normal values (ULN) of transglutaminase antibody (TGA) concentrations in ELISA. The error bars represent the higher and lower limits of 95% confidence intervals of sensitivity at a given ULN. ** *p* = 0.0051, *** *p* < 0.001.

We also tested all these samples in 1:2.5 serum dilutions because the removal of cells resulted in a very low background at the staining and allowed us to apply more concentrated serum samples. The serum samples of 62 CD patients negative for conventional EMA results at diagnosis were selected from our biobank for testing at both 1:2.5 and 1:10 serum dilutions. A clear positive EMA reaction was seen in 1:10 dilutions in 16 samples and in 1:2.5 dilutions in 12 more samples on the decellularized biomatrix, thus altogether in 28 (45%) of the 62 CD samples, indicating a markedly improved sensitivity. All 105 control samples were negative for the EMA reaction at 1:2.5 dilutions (two control samples showed punctate IgA staining at cell anchor site remnants, but no EMA pattern). The sensitivity values obtained in the combined retrospective and prospective CD cohorts after retesting the three initially negative CD samples from the prospective cohort at 1:2.5 dilutions are presented in Figure 5. (The prospectively tested samples positive on the biomatrix at 1:10 serum dilutions were regarded as positive without retesting at 1:2.5.)

In summary, the biomatrix allowed for significantly more sensitive detection of EMA at low positive (1-3xULN) TGA antibody concentrations without the loss of specificity.

### 2.5. Detection of Both IgA and IgG Antibodies

**Figure 6:**
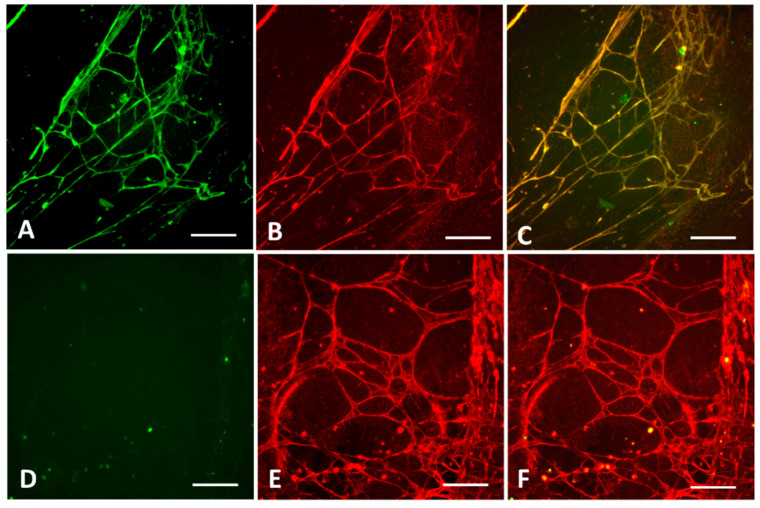
Binding of an IgA-competent (**A**–**C**) and of an IgA-deficient (**D**–**F**) celiac serum sample to decellularized biomatrix. Panels (**A**,**D**) show the staining with fluorescein isothiocyanate-labeled anti-human IgA secondary antibodies in green, and panels (**B**,**E**) with polyclonal rabbit anti-human IgG secondary antibodies and AlexaFluor 568-conjugated goat anti-rabbit antibodies in red. On panel (**C**), positive IgA and positive IgG stainings merge to yellow, while for the IgA-deficient patient, no IgA staining is visible and thus, the merged IgA and IgG results (**F**) remain red. Bars represent 50 µm.

The decellularized HUVEC biomatrix also gave less background and a well-evaluable positivity pattern for detecting EMA-IgG antibodies in the five tested samples from untreated CD patients with IgA deficiency, whereas the IgA-deficient controls were negative. Combining EMA-IgA and IgG detection on the same biomatrix well was also possible by using green fluorescent labeling for IgA and red for IgG antibodies (Figure 6). This double staining may recognize IgA-deficient CD patients also in the absence of the total serum IgA measurement and when EMA-IgA is negative.

### 2.6. Interobserver Variation

Fifty samples were evaluated separately by an experienced and an untrained evaluator. The untrained evaluator judged 48 samples as positive or negative in agreement with the experienced evaluator and two negative samples as dubious or positive, thus, agreement was high (κ = 0.92).

## 3. Discussion

The EMA test was introduced in 1984 for the detection of antibodies in CD and it is still a highly appreciated assay due to its easy procedure and excellent specificity [7,20] in experienced laboratories. Currently, it is mainly applied as a second, confirmatory test, and forms part of the ESPGHAN 2020 criteria [2] for the non-biopsy diagnosis of CD in children and adolescents, and of equivalent guidelines also for adults in some countries, e.g., Finland. For this reason, the lack of available tissue substrates may compromise the adherence to guidelines in the everyday diagnostic workup. Here, we present a cell-assembled biomatrix that can be applied with similar positive predictive value as the conventional tissue-based EMA testing, and it has the potential for unlimited supply and contains human antigens.

**Figure A1:**
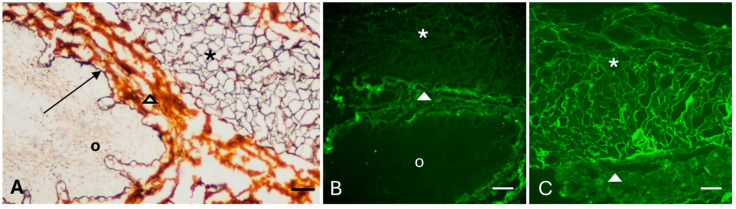
Endomysial and connective tissue parts of a monkey esophagus have different collagen expression and celiac antigenicity. (**A**) Silver staining demonstrates positivity for the endomysial structures (black) in the muscular layer (asterisk) and for the reticulin lining under the epithelium (arrow). The silver staining-positive structures correspond to the structures recognized by celiac disease IgA in the EMA test (**C**). The submucosal connective tissue (triangles) was neither antigenic for celiac patient samples, nor gave a positive staining in the silver impregnation (remains brown) and corresponded to collagen I expression in immunofluorescence (**B**). Symbols: asterisks indicate endomysial layers, triangles indicate collagen I-rich non-endomysial connective tissue, and circles indicate the epithelium of the esophagus. Bars represent 50 µm.

We have shown by the classical silver staining that the biomatrix produced in this study is highly organized into a honeycomb pattern argyrophilic network characteristic of tissue endomysium [16,21]. Endomysial-type tissues are distinct from common connective tissues produced by fibroblasts, which only give brown color with the silver staining when endomysial structures stain to black. In fact, the connective tissue parts of the monkey esophagus, e.g., the submucosa, built up of mainly type-I collagens, are not antigenic for CD antibodies (Figure A1). Collagen I is the predominant fibrous component in the extracellular matrix of most conventionally cultured cells, including fibroblasts from the umbilical cord [18]; this can explain why fibroblasts were not good candidates to produce the endomysial-type matrix. In fact, fibroblasts were seen to produce high amounts of TG2 in its antigenic form for CD antbodies, but the antigen remained within the cells or was less efficiently externalized and in a form that did not build up a well-recognizable network pattern. The same was observed for fibronectin, the main anchor protein for TG2 in the extracellular matrix (Figure S1H). Carvalho and coworkers [22] also noted important differences between HUVECs and other mesenchymal cells in the composition of their extracellular matrix, notably collagen I and fibronectin expression patterns, when they evaluated their osteogenic properties. It is, thus, conceivable that the special TG2-rich composition of the endomysial extracellular tissue is the result of a different matrix production pathway compared to that fibroblasts commonly perform when they are building up common connective tissues.

In contrast, endothelial cells are known to develop argyrophilic structures when they form vessels [23]. HUVECs stained positive for VE-cadherin in the mature biomatrix, thus retaining their endothelial character. In addition, VEGF was able to promote the formation of the antigenic biomatrix in our experiments. Vessels are the targets of celiac antibody deposition in vivoin patients [11,24,25,26] and have been shown to mediate the biological effects of celiac antibodies in experimental models both in vivoand in vitro[27,28,29].

However, the mature fibrillar network we obtained by culturing HUVECs in the described way seemed structurally different from the well-known tubule formation of endothelial cells [11,29] and was completely extracellular. Further, no cords of cells were observed. Nonetheless, it cannot be excluded that the early phases of HUVEC differentiation during the initial 24–48 h in EGM-2 were common with the pathway of tubule formation, which, however, continued in a different direction after the medium change. The absence of externally added extracellular matrix support probably also made a difference. We observed that the medium change from EGM-2 (contains 2% FBS) to Medium 199 with 10% FBS and 10% EGM-2 was important to promote the externalization of TG2 into the fibrillar network. In addition, the higher FBS content is known to help cells produce more matrix components, while the low FBS concentration may induce differentiation. On the other hand, EGM-2 was found necessary at the seeding for the proper adhesion of the cells to the chambers that had no precoating with biomolecules.

The predominance of collagen III and collagen IV both in the HUVEC-ECM and tissue endomysium indicates that they represent similar fibrillar structures, reticulin [30]. Reticulin, thus reticular fibers, were the first identified antigenic structures for CD autoantibodies described as early as 1971 [3]. Later studies showed that the celiac autoantigen in both the endomysium and reticulin is extracellular TG2, and CD antibodies do not bind to either the endomysium or reticulin in tissues from TG2 ^−/−^ mice [31] or after the removal of TG2 by chemicals [8]. The exact biochemical composition of endomysium or reticulin has never been determined due to the complexity of post-translational modifications of those fibers and of the attached glycosylated molecules [30]. Endomysium is, thus, hitherto defined only anatomically or by the silver impregnation method [32]. This is why we felt it more informative to examine the biomatrix rather by immunofluorescent pattern analysis instead of blots, which gave contradictory results on reticulin in earlier studies. In addition, Western blots also have very restricted usefulness for the celiac autoantigen [13], because mainly conformational epitopes of TG2 are targeted by the patient samples [11].

Our prospective pilot study demonstrates the high diagnostic performance of the biomatrix-based new EMA test in a high-pretest probability setting of a tertiary center but also shows that confirmatory testing is indeed needed. Almost 20% of the patients prospectively enrolled with a positive celiac antibody result at the referral site turned out to be negative in our testing. The EMA test result has importance not only in the non-biopsy diagnostic approach of CD when the TGA-ELISA results are highly positive, but also for the decision to make a duodenal biopsy when the TGA-ELISA result is low positive, borderline, or dubious. The initial histology result may be negative in such patients, but a final CD diagnosis in the future is more probable in EMA-positive than in EMA-negative cases [33]. The EMA-positive result is, thus, important to define a case as potential celiac disease, which requires a regular clinical follow-up. In addition, the EMA test identified in several case findings and population screening studies new CD cases that were TGA-ELISA-negative [34].

EMA positivity indicates that the coeliac-relevant epitopes of TG2 are targeted by the investigated serum antibodies. In contrast, TGA positivity only means a reaction with TG2 in general, which may or may not be [11] CD-related. The HUVEC-ECM exposes in the same way the epitope-2 of TG2 as the EMA tissues because the TG2 antigen is bound to fibronectin in both. TG2 has a specific binding site for fibronectin, and when in complex with it, non-specific antibodies sticking to non-coeliac TG2 epitopes are less likely [11,13]. This makes the EMA test result highly specific for CD. It also contributes to the specificity that, in the final format, the biomatrix is decellularized and contains only extracellular matrix components with antigenic TG2 and the obtained binding pattern is, thus, free from disturbing non-specific reactivity to cell bodies. Recombinant TG2 proteins utilized in ELISA and other immunoassays as antigens have variable quality and are not always true imitators of the natural autoantigen due to poor folding and even mismatching amino acid sequences [14]. Currently, no quality control is in force for recombinant TGs, although it is known that proteins produced in *E. coli* have less stable conformation than those made in insect cells.

IgA deficiency is a further challenge for CD antibody detection, and it is often not known at the initial testing [2,3]. Contrary to the ESPGHAN guidelines, which suggest first the measurement of the total serum IgA level [2], many clinical laboratories offer both IgA and IgG TGA and/or DGP determinations in combination. These IgG tests are less well optimized than the IgA-TGA tests, and more often yield elevated results in the absence of CD [2,35,36]. Moreover, healthy children, investigated in a prospective study, were shown to regularly develop high levels of IgG DGP antibodies in response to gluten [5]. Nowadays, patients with isolated positivity for IgG-class TGA or DGP antibodies constitute an increasing bulk for confirmatory testing in tertiary centers, as we also experienced this in our prospective patient group. IgG-class EMA positivity is highly predictive for CD in IgA-deficient subjects and represents a valid indication for biopsy [4,12], although alternative tissue substrates, such as the umbilical cord of premature babies, human appendix [15], jejunum [4], or liver [21], are better suited for the IgG EMA testing than monkey esophagus because the presence of reticulin fibers ease the visual evaluation. The biomatrix, built up of a reticulin network and having a very low background, may have value for IgG EMA detection in the same step as IgA EMA using the double staining presented in this study. Further studies are needed to determine the diagnostic accuracy of this approach on a larger patient group in direct comparison with IgG DGP determinations also in use for the evaluation of IgA-deficient patients [36].

The EMA test has been criticized as being laborious and having observer-dependent and less sensitive results than ELISA or automated immunoassays. Further, as a microscopic test, it is not welcomed by laboratories with a high degree of automatization. The use of presently developed biomatrix may improve both the availability and applicability of EMA testing. The biomatrix is produced by monolayer cell culture, thus there is no need to cut sections. The removal of the cells before use can decrease the ethical concerns associated with tissue substrates. Decellularization also significantly improved sensitivity and made possible the reading of the test result even without previous training. Since the biomatrix itself is antigenic and cell bodies generate no background, any network-like positivity seen indicates an EMA reaction. A degree of co-localization evaluation assisted by a computer by using a counterstain for TG2 (Figure 3 and Figure 4) or applying artificial intelligence for image analysis can be a possibility as well in the future.

The endothelial cells producing the biomatrix are commercially available and can be easily grown on a large scale with relatively low costs covering plasticware and culture media. One bottle of EGM-2 might be sufficient for preparing 3000–4000 biomatrix wells with some additional low-cost media. Currently, the cost for commercially available monkey esophagus slides varies between EUR 5 and 10 per section in Europe. The evaluation of one positive patient serum sample may require several sections for the semiquantitative determination of antibody concentration by titration from serial dilutions, although many laboratories use only one or two dilutions. Alternatively, laboratories in large clinical centers cut frozen sections from umbilical cords or order them from the pathology departments. Hospitals, however, may not have access to cell culture facilities, and the actual costs for setting up a biomatrix-based EMA testing may differ in different clinical settings and countries, though costs are estimated to be much lower than obtaining commercial or frozen tissue slides for the EMA testing. Since the biomatrix slides fixed in methanol can be stored and distributed easily, centralized production in biomedical labs or university centers also may be an option.

## 4. Materials and Methods

### 4.1. Cell Culture

The HUVECs were obtained from Lonza (Basel, Switzerland) or prepared from anonymously donated human umbilical cords by collagenase digestion as described by Palatka et al. [37]. The cells were maintained at 37 °C with 5% CO_2_ in EGM-2 Endothelial Growth Medium with added Single Quots ^TM^ (CC-3156 and CC-4176, Lonza, Basel, Switzerland), or in Medium 199 (HyClone, Logan, UT, USA) supplemented with 10% (*v*/*v*) fetal bovine serum (Merck-Millipore, Darmstadt, Germany), 20 mM HEPES, 100 U/mL Penicillin, 100 mg/mL Streptomycin, 2.5 mg/mL Amphotericin B (all from Biosera, Nuaille, France), and optionally adding 10% (*v*/*v*) complete EGM-2. The cells were immortalized by the viral delivery of the telomerase gene using pBABE-neo-hTERT (gift from Bob Weinberg, Addgene #1774 (Addgene repository, Watertown, MA, USA) [38] as earlier described [19]. Transfected cells were selected using 300 µg/mL G418 (Merck-Millipore, Darmstadt, Germany). Immortalized cells completely retain the morphological properties of primary endothelial cells and express von Willebrand factor, CD31, and TG2 [39].

The fibroblasts and myofibroblasts were prepared by trypsin digestion from Wharton’s jelly and the vessel walls of the umbilical cord and cultured as described by Sulkanen et al. [16].

### 4.2. Celiac Antibody Detection on Biomatrix

For the evaluation of celiac antibody binding, the HUVECs were seeded in subconfluent densities to 18-well µ-Slide chamber slides (Ibidi, Gräfelfing, Germany) manufactured from acetone-resistant plastic and cultured for 3–6 days. The chambers were fixated in precooled acetone or in a solution with 90% (*v*/*v*) methanol and 10% (*v*/*v*) MES buffer (100 mM 2-(4-morpholino)-ethanesulfonic acid with 1 mM EGTA and 1 mM MgCl_2_, pH 6.9) at −20 °C for 10 min with prior and after washings in phosphate-buffered saline, pH 7.2 (PBS). In some experiments, the cells were removed from the slides with 0.1% sodium deoxycholate in PBS before the fixation. Serum samples diluted in PBS were added for 30 min at room temperature. Initially, we used 1:10 serum dilutions, because 100 µL solution (four times more than usually used for individual tissue sections) was needed to fill a chamber, and in this way, we applied approximately similar amounts of antibodies per specimen as when using 1:2.5 serum dilutions on tissues. In later measurements, the serum samples were tested in both 1:2.5 and 1:10 dilutions. After washings with PBS, bound IgA antibodies were visualized with fluorescein isothiocyanate (FITC)-labeled rabbit anti-human IgA polyclonal antibodies (Dako, Roskilde, Denmark) and evaluated with an Olympus CKX41 inverted fluorescent microscope as positive (visually graded 1–3) or negative. The staining of cell bodies was not regarded as a positive result. The samples for the diagnostic evaluation were tested in a blinded fashion for the results of other antibody tests and biopsies. Fifty samples were evaluated separately by an experienced observer and an untrained evaluator.

Double-immunofluorescent labeling for both bound IgA and IgG celiac antibodies was performed by adding the secondary antibodies in the order (1) rabbit antibodies against human IgG (Dako) followed by (2) Alexa Fluor 568-labeled anti-rabbit goat antibodies (Thermo Fisher Hungary, Budapest, Hungary) and (3) FITC-labeled rabbit anti-human IgA antibodies (Dako, Roskilde, Denmark), with PBS washings between these steps to avoid cross-reactions.

The binding of the cloned celiac patient-derived antibodies [11] selectively targeting the celiac epitope 2 of TG2 was recognized by anti-V5 tag monoclonal mouse antibodies (Thermo Fisher Hungary, Budapest, Hungary) followed by Alexa Fluor 488 conjugated secondary anti-mouse antibodies (Thermo Fisher Hungary, Budapest, Hungary).

### 4.3. Microscopic Analysis of Biomatrix and Tissue Proteins

Slides with the prepared biomatrix or monkey esophagus sections were also stained with mouse monoclonal antibodies to TG2 (CUB7402) diluted 1:200 in PBS, or with the primary antibodies listed in Table 2 followed by AlexaFluor 568 or 488 conjugated goat antibodies (Thermo Fisher Hungary, Budapest, Hungary) to rabbit or mouse immunoglobulins, as appropriate.

Silver impregnation staining for defining endomysial structures was performed according to the modification of the original Gömöri’s method described by Krutsay [32]. The cells were seeded to glass slides for the silver staining because plastic was incompatible with the used reagents.

### 4.4. Patient Samples

During the development and optimization, serum samples from three IgA-competent CD patients with positive anti-transglutaminase antibodies (TGAs), EMA antibodies, and Marsh III histology in the small bowel, and from three non-celiac patients with normal small bowel histology were used.

To assess the sensitivity, specificity, and diagnostic accuracy of EMA tested on the prepared HUVEC biomatrix, serum samples were collected between May–September 2023 at the Celiac Disease Center, Heim Pál National Pediatric Institute, Budapest, and tested prospectively from 90 patients (median age: 6.3 years, range 2.4–40) consecutively referred for CD evaluation because of previous positive TGA or gliadin antibody laboratory results at the referral site or detected by various rapid home tests. Details on demographic data, the initial antibody tests, and the clinical symptoms leading to the testing are presented in Table S2. All these patients were evaluated with standard hospital TGA ELISA and EMA tests on monkey esophagus, human umbilical cord, and human appendix substrates for IgA-class celiac antibodies as a part of their diagnostic workup from the same sample which was used and number-coded for the evaluation on the HUVEC biomatrix upon informed consent. None of these patients were IgA deficient, but if the test before referral had been positive only for IgG-class celiac antibodies, TGA-IgG and EMA-IgG tests were also performed. The diagnosis of CD was established according to the ESPGHAN 2020 guidelines [2] by histology or by the non-biopsy route as appropriate.

A second, retrospective cohort (median age: 9.3 years, range 3–21) of 62 CD patients with Marsh II-III lesions and positive TGA-IgA but negative EMA-IgA results on tissues and of 105 non-celiac control patients with normal histology were included to assess sensitivity and specificity further using the decellularized HUVEC biomatrix.

Serum samples from five IgA-deficient CD patients with negative IgA but positive IgG TGA and EMA antibodies having Marsh III histology and three non-celiac IgA-deficient seronegative controls were also tested.

### 4.5. Clinical Antibody Testing

The TGA levels were measured by a red blood cell transglutaminase-based sandwich ELISA described earlier [40] and validated in the ProCeDe international study [12]. In brief, the TG2 antigen was captured to the ELISA plate coated with antibodies to TG2, followed by the incubation with patient serum samples diluted 1:100 and peroxidase-conjugated anti-human IgA (Dako, Roskilde, Denmark). The results were calculated from a 4-parameter fit curve utilizing 6 calibrators (0, 3, 7, 16, 40, 100 U/mL) adjusted to calibrator values of the Phadia Celikey Varelisa test (Phadia, Uppsala, Sweden). The cut-off for positivity (1xULN) was 3 U/mL.

EMA testing on the tissues was performed by the indirect immunofluorescent method with serum diluted to 1:2.5 using unfixed frozen sections cut from a composite block containing monkey esophagus, human umbilical cord, and human appendix tissues. All the patient samples were evaluated on all three tissues. The results were regarded as positive when any of these substrates showed IgA binding around endomysial structures in conjunction with the classical additional components of the EMA binding (staining of subepithelial fibers and of those in lymphoid follicles in the esophagus and appendix, and positivity of Wharton’s jelly fibroblasts in the umbilical cord).

### 4.6. Statistical Analysis

Diagnostic parameters were calculated using the GraphPad Prism 8.0.1. software (Dotmatics, Boston, MA, USA). Diagnostic sensitivity was compared by the Mann–Whitney test, *p* values < 0.05 were regarded as significant. The 95% confidence intervals were determined by the Wilson method.

## 5. Conclusions

The EMA test is still diagnostically important, especially in children, for the safe diagnosis of CD without invasive endoscopy and for the evaluation of unclear cases with low antibody positivity. Cell-assembled biomatrix after decellularization offers a reliable, easy-to-read, and ethically acceptable alternative for monkey esophagus and human umbilical cord in EMA testing.

## 6. Patents

The University of Debrecen submitted a patent application based on the results of this study.

## Figures and Tables

**Table 1:** Diagnostic performance of the EMA testing on the HUVEC-ECM biomatrix, on conventional tissue sections, and of the ELISA detection of coeliac antibodies in the prospectively investigated patient cohort.

	EMA-IgA+ on HUVEC-ECM	EMA-IgA− on HUVEC- ECM	EMA-IgA+ on Tissues	EMA-IgA− on Tissues	TGA-IgA ELISA+	TGA-IgA ELISA−
Celiac disease (n = 68)	65	3	62	6	68	0
No celiac disease (n = 6)	0	6	0	6	2	4
Total with final diagnosis (n = 74 †)	65	9	62	12	70	4
Sensitivity (%)	95.6 (92.7–98.5)		91.2 (87.8–94.5)		100	
Specificity (%)	100		100		66.7 ‡	
Positive predictive value (%)	100		100		97.1	
Negative predictive value (%)	66.7		50.0		100	
Diagnostic accuracy (%)	95.9		91.9		97.3	

† only the results of patients with biopsy results or qualifying for the non-biopsy coeliac disease diagnosis were considered for calculating the diagnostic performance parameters. ‡ most patients with negative results did not undergo biopsy and thus were not in the calculation. EMA, endomysial antibodies; TGA, transglutaminase antibodies; HUVEC-ECM, extracellular biomatrix produced by human umbilical cord vein endothelial cells; 95% confidence intervals are shown in brackets.

**Table 2:** Primary antibodies used for the immunofluorescent studies.

Target Antigen	Antibody Type	Clone	Manufacturer	Catalog Number
Collagen I	mouse monoclonal	C11	Chemicon, Melbourne, Australia	MAB1340
Collagen III	mouse monoclonal	IE7-D7	Chemicon, Melbourne, Australia	MAB3392
Collagen IV	mouse monoclonal	23IIC3	Chemicon, Melbourne, Australia	MAB1910
Cytokeratin-19	mouse monoclonal	A-3	SantaCruz, Heidelberg, Germany	sc378126
Fibronectin	rabbit polyclonal	-	Sigma/Merck, Darmstadt, Germany	F3648
Laminin	rabbit polyclonal	-	Sigma/Merck, Darmstadt, Germany	L9393
Lpp	mouse monoclonal	LPP4	Sigma/Merck, Darmstadt, Germany	L2920
Transglutaminase-2	mouse monoclonal	CUB7402	NeoMarkers, Fremont, CA, USA	CUB7402
VE-cadherin	rabbit monoclonal	048	Invitrogen, Carlsbad, CA, USA	MA-29141
Vinculin	rabbit polyclonal	-	Sigma/Merck, Darmstadt, Germany	HPA002131

Lpp, Lipoma preferred partner protein.

## Data Availability

Data are available from the authors upon reasonable request.

## Supplementary Materials

The following are available online at www.mdpi.com/article/10.3390/ijms26031012/s1.

## Abbreviations

CD	celiac disease
DGP	deamidated gliadin peptide antibodies
ECM	extracellular matrix
EGM-2	endothelial cell growth medium 2
ELISA	enzyme-linked immunosorbent assay
EMA	endomysial antibody
FBS	fetal bovine serum
FITC	fluorescein isothiocyanate
HUVEC	human umbilical vein endothelial cell
PBS	phosphate-buffered saline
TG2	type-2 transglutaminase (also called tissue transglutaminase or tTG)
TGA	anti-transglutaminase antibody
