# Supplementary material for: Development of Cell-Assembled Human Endomysial-Type Biomatrix Substrate for the Detection of Celiac Disease Autoantibodies
Source: Int J Mol Sci. 2025 Jan 24;26(3):1012. doi: 10.3390/ijms26031012 (PMC11817494; doi:10.3390/ijms26031012)
Supplement: Supplementary file 1 [file ijms-26-01012-s001.zip › ijms-3431662-supplementary.pdf]

SUPPLEMENTARY FILES

Table S1. Effect of culturing time and growth media on the formation of HUVEC-ECM matrix antigenicity for celiac disease antibodies

Culture time	Medium	Sample 1 (pos)	Sample 2 (pos)	Sample 3 (pos)	Sample 4 (borderline)	Sample 5 (borderline)	Sample 6 (neg)
4 days	EGM-2	- [(a)]	- [(a)]	- [(a)]	-	-	-
	Medium199 + 10 V/V% FBS and 10 V/V% EGM-2	+++	+++	+++	+	-	-
	Medium199 + 10 V/V% FBS	-	-	-	-	-	-
5 days	EGM-2	++	++	++	+	-	-
	Medium199 + 10 V/V% FBS and 10 V/V% EGM-2	+++	+++	+++	+	+/-	-
	Medium199 + 10 V/V% FBS	-	-	-	-	-	-
6 days	EGM-2	+++	+++	+++	+	-	-
	Medium199 + 10 V/V% FBS and 10% EGM-2	+++	+++	+++	+	-	-
	Medium199 + 10 V/V% FBS	+	-	-	+	-	-

(a) intracellular positivity

HUVEC-ECM: antigenic extracellular matrix produced by human umbilical cord vein endothelial cells, FBS, fetal bovine serum,

Serum 1-6, patient samples diluted 1:10 in phosphate-buffered saline

Table S2. Initial characteristics of the patients included in the prospective clinical study

	Prospective cohort (n=90)
Median age, ranges (years)	6.4 (2.4-40)
Male/Female	30/60
Clinical symptoms leading to the first celiac antibody testing	
Enteral symptoms (diarrhea, lactose intolerance, bloating)	18
Recurrent abdominal pain	22
Constipation	5
Retarded growth, failure to thrive or weight loss	3
Tiredness, headaches, hair loss, nonspecific rash	10
Accidental finding (irrelevant or no symptoms)	2
Type-1 diabetes or thyroid disease	4
First-degree family members with or without symptoms	23
Population screening by rapid test	3
Type and outcome of the first celiac antibody test	
TGA-IgA positive laboratory result	48
TGA-IgG positive laboratory result with negative TGA-IgA	8
TGA-IgA+IgG positive laboratory result with blended test	2
TGA-IgA positive rapid test result	14
DGP positive laboratory or rapid test result	7
Not specified	1
Median time interval between the first and the study antibody testing, range (weeks)	3.3 (1-30)

TGA, antibodies to transglutaminase 2, DGP, antibodies to deamidated gliadin peptides

Table S3. Comparison of EMA results obtained using the HUVEC-ECM substrate with results of conventional diagnostic tests for celiac disease in the prospectively tested cohort

TGA-IgA ELISA		EMA-IgA+ on tissues	EMA-IgA+ on HUVEC-ECM	Final CD diagnosis by ESPGHAN 2020 criteria
Concentration	n	n	n	n
≥10 xULN	51	51	51	51
2-9.9 xULN	16	11	14	15
1-1.9 xULN	6	0	2	2
<1 xULN	17	0	0	0
Total	90	62	67	68

HUVEC-ECM, extracellular biomatrix produced by human umbilical cord vein endothelial cells, TGA, antibodies to transglutaminase 2, EMA, endomysial antibodies, CD, celiac disease, ULN, upper limit of normal (3 U/ml), n, number of cases

Figure S1.

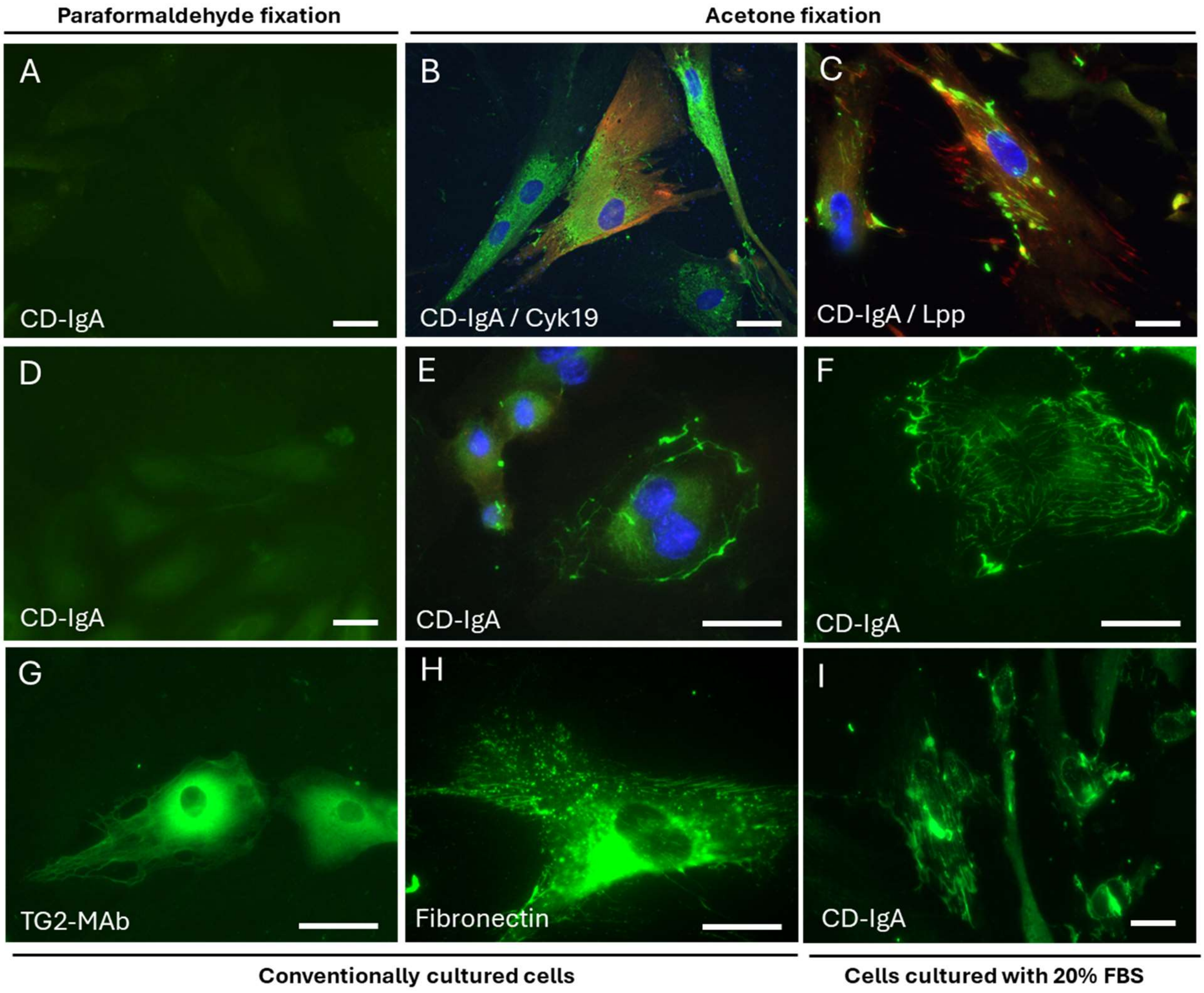

Figure S1. Detection of transglutaminase 2 in cultured cells and in their extracellular matrix

Pictures were taken on human umbilical cord vein-derived myofibroblasts (A-C), endothelial cells (D-G) and Wharton's jelly-derived fibroblasts (H-I) stained with celiac disease patient serum (CD-IgA), CUB7402 monoclonal antibodies to transglutaminase 2 (TG2-MAb) or rabbit antibodies to fibronectin followed by fluorescein isothiocynate(FITC)-labeled anti-human IgA or AlexaFluor 488-labeled secondary antibodies generating a green fluorescent signal. Myofibroblasts were also stained for smooth muscle cell markers cytokeratin-19 (Cyk19) and lipoma preferred-partner protein (Lpp) using AlexaFluor 568-labeled secondary antibodies generating a red signal. Cell nuclei were stained to blue with DAPI. Bars represent 10 µm.

Contrary to CUB7402, CD-IgA antibodies were not able to recognize the TG2 antigen after paraformaldehyde fixation, but showed a positive signal after acetone fixation.

Endothelial cells deposited the TG2 antigen to their extracellular matrix in an organized fashion (E), while myofibroblasts and fibroblasts showed bright intracellular TG2 positivity, but little externalization (B) under conventional culturing conditions, with a similar pattern for fibronectin (H). High fetal bovine serum concentration (20%) in the culture media enhanced TG2 externalization in all three cell types (C,F,I). However, TG2 in the matrix of fibroblasts and myofibroblasts appeared in irregular, big clusters without a clear, diagnostically useful pattern.

FBS, fetal bovine serum

Figure S2.

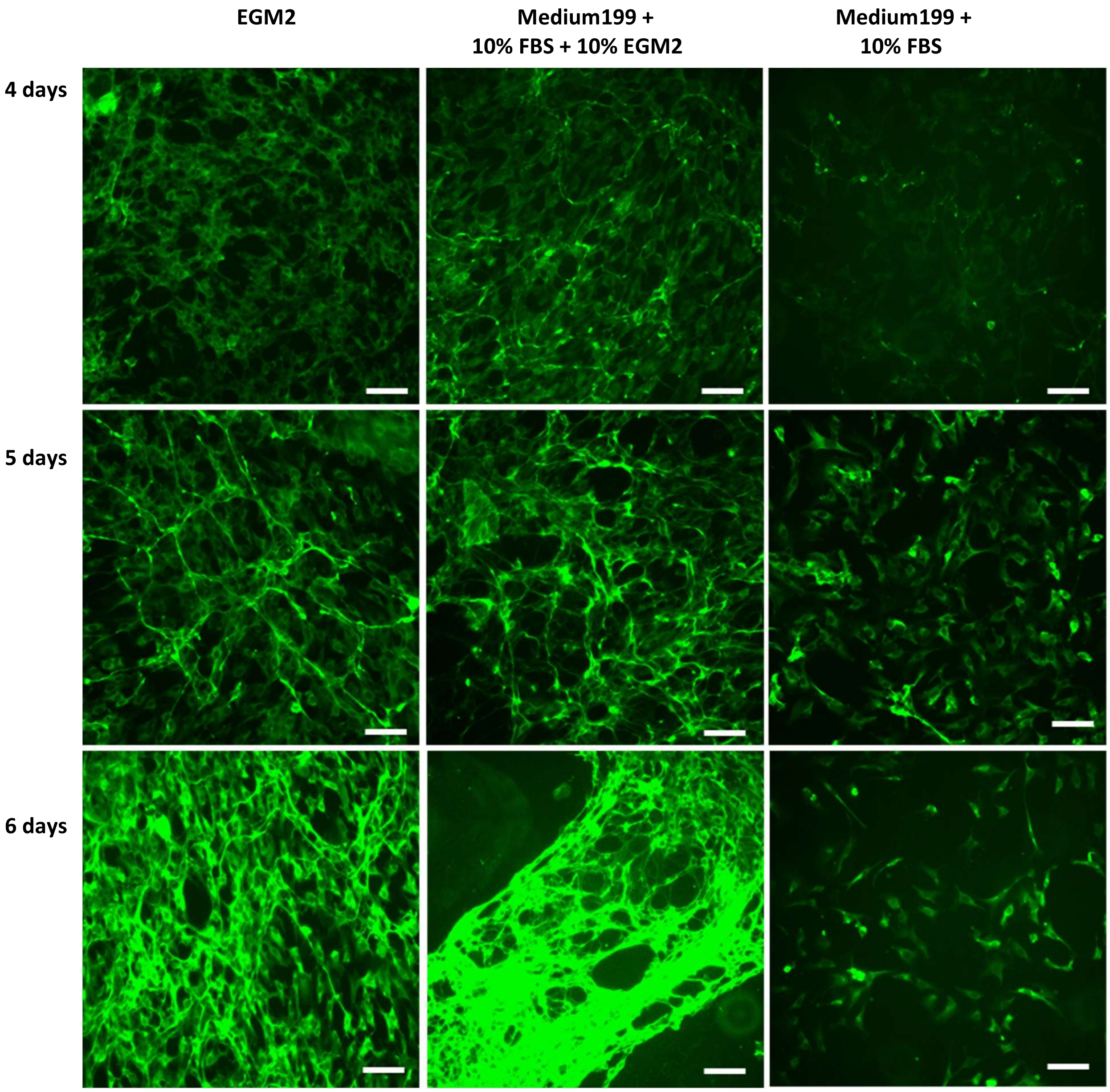

Figure S2. Development of the celiac antigenic extracellular matrix in HUVEC cell cultures using different culture media

Cells were grown in the indicated culture media for 4-6 days, then the cultures were terminated by acetone fixation and stained with celiac patient serum. Bound IgA antibodies were detected by fluorescein isothiocyanate-conjugated secondary antibodies. The images were taken with the same exposure times. Bars represent 50 µm.

HUVEC, human umbilical cord-derived endothelial cells, FBS, fetal bovine serum

Figure S3.

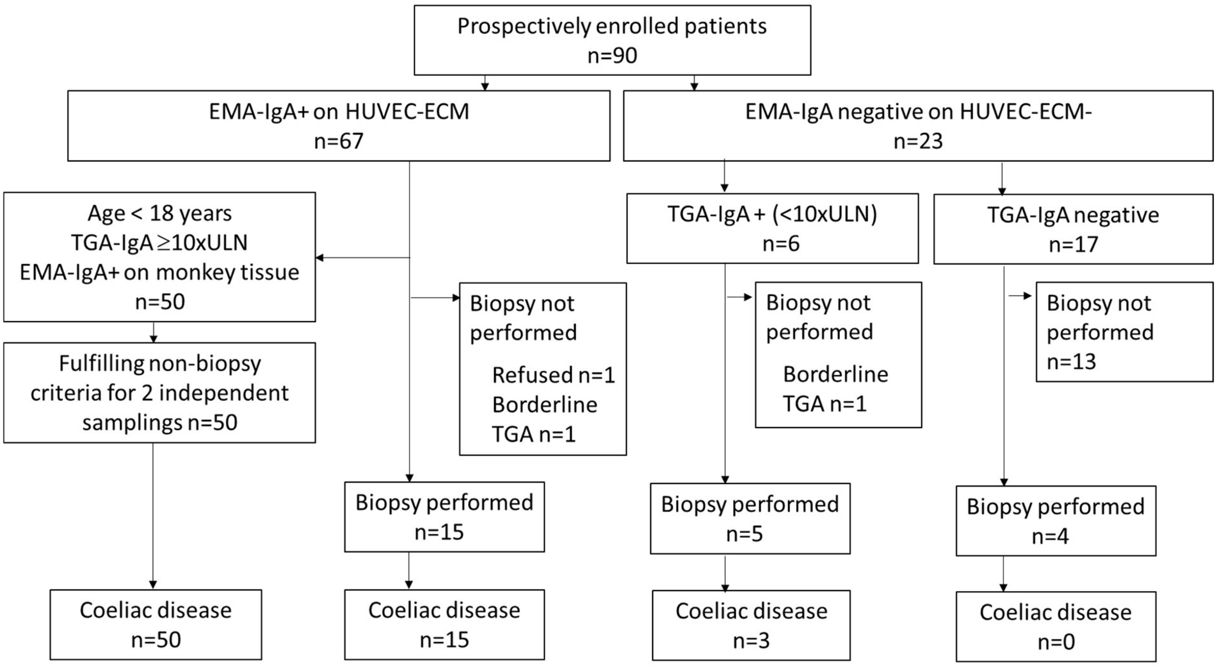

Figure S3. Flowchart of the prospective clinical study
